# Phylogenomic Coalescent Analyses of Avian Retroelements Infer Zero-Length Branches at the Base of Neoaves, Emergent Support for Controversial Clades, and Ancient Introgressive Hybridization in Afroaves

**DOI:** 10.3390/genes13071167

**Published:** 2022-06-28

**Authors:** John Gatesy, Mark S. Springer

**Affiliations:** 1 Division of Vertebrate Zoology, American Museum of Natural History, New York, NY 10024, USA; 2 Department of Evolution, Ecology, and Organismal Biology, University of California, Riverside, CA 92521, USA; mark.springer@ucr.edu

**Keywords:** bird, owl, transposon, multispecies coalescent, gene tree, incomplete lineage sorting, species tree

## Abstract

Retroelement insertions (RIs) are low-homoplasy characters that are ideal data for addressing deep evolutionary radiations, where gene tree reconstruction errors can severely hinder phylogenetic inference with DNA and protein sequence data. Phylogenomic studies of Neoaves, a large clade of birds (>9000 species) that first diversified near the Cretaceous–Paleogene boundary, have yielded an array of robustly supported, contradictory relationships among deep lineages. Here, we reanalyzed a large RI matrix for birds using recently proposed quartet-based coalescent methods that enable inference of large species trees including branch lengths in coalescent units, clade-support, statistical tests for gene flow, and combined analysis with DNA-sequence-based gene trees. Genome-scale coalescent analyses revealed extremely short branches at the base of Neoaves, meager branch support, and limited congruence with previous work at the most challenging nodes. Despite widespread topological conflicts with DNA-sequence-based trees, combined analyses of RIs with thousands of gene trees show emergent support for multiple higher-level clades (Columbea, Passerea, Columbimorphae, Otidimorphae, Phaethoquornithes). RIs express asymmetrical support for deep relationships within the subclade Afroaves that hints at ancient gene flow involving the owl lineage (Strigiformes). Because DNA-sequence data are challenged by gene tree-reconstruction error, analysis of RIs represents one approach for improving gene tree-based methods when divergences are deep, internodes are short, terminal branches are long, and introgressive hybridization further confounds species–tree inference.

## 1. Introduction

**Figure 1:**
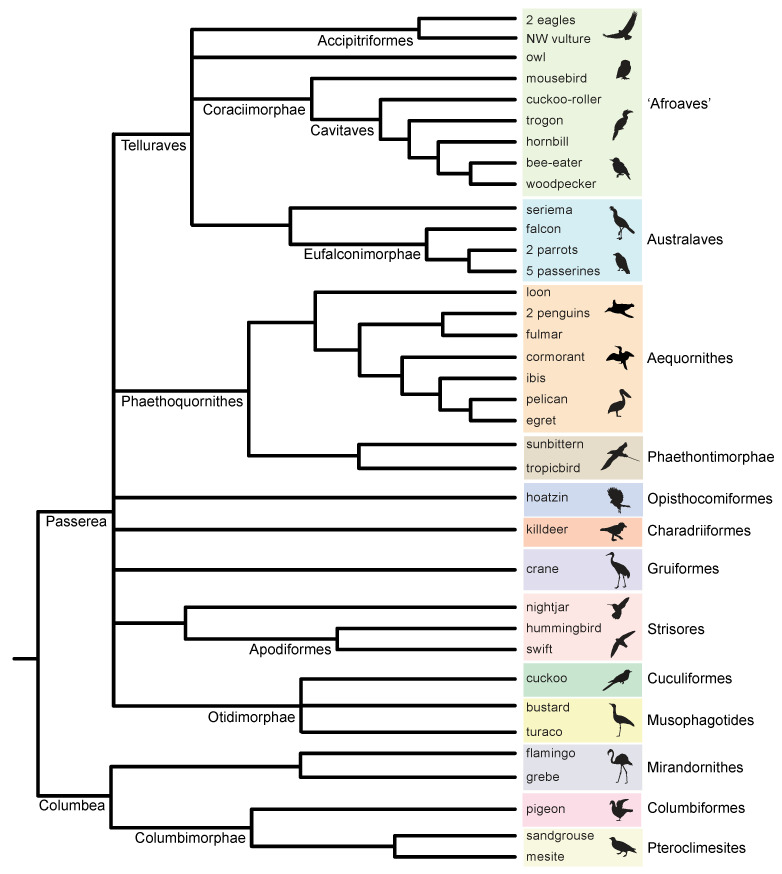
Informal consensus species tree for Neoaves based on three recent summaries [3,4,8]. Terminal taxa indicate genomes analyzed by Jarvis et al. [1], and higher-level taxa are shown at internodes and to the right of terminals (delimited by color). Three clades, Afroaves, Musophagotides, and Coraciimorphae + Strigiformes (owls) are not resolved in the consensus tree but were featured in the summaries of Reddy et al. [3], Houde et al. [8], and Braun et al. [4], respectively. ‘NW vulture’ = New World vulture. Silhouettes are from http://phylopic.org/ (accessed on 1 May 2020).

The early radiation of Neoaves, the clade that includes all extant birds except Palaeognathae (ratites, tinamous) and Galloanserae (land- and water-fowl), has defied convincing systematic resolution. The early diversification of the group near the Cretaceous–Paleogene boundary exemplifies the challenges that confront genome-scale analysis when the splitting of evolutionary lineages is rapid and deep in time [1,2]. Phylogenomic approaches have generated strong topological conflicts when different datasets and contrasting analytical protocols have been applied, with the diversity of results perhaps leading to more confusion than resolution of this challenging phylogenetic puzzle [3,4]. One view is that the base of Neoaves represents a ‘hard polytomy’ that should not be represented by a strictly bifurcating tree with relationships better expressed as a reticulating network or a near-simultaneous starburst of eight, or more, deep lineages [5,6]. An alternative perspective represents an emerging consensus (Figure 1) based on congruence among analyses of non-coding DNA sequences for some higher-level relationships, with conflicts to trees based on protein-coding sequences that have been attributed to shifts in nucleotide-base composition, functional convergence, homology errors in the underlying sequence alignments, long-branch effects, and/or poor fit to standard nucleotide substitution models that hinder the downstream inference of the overall species tree [1,3,4,7,8].

**Figure 2:**
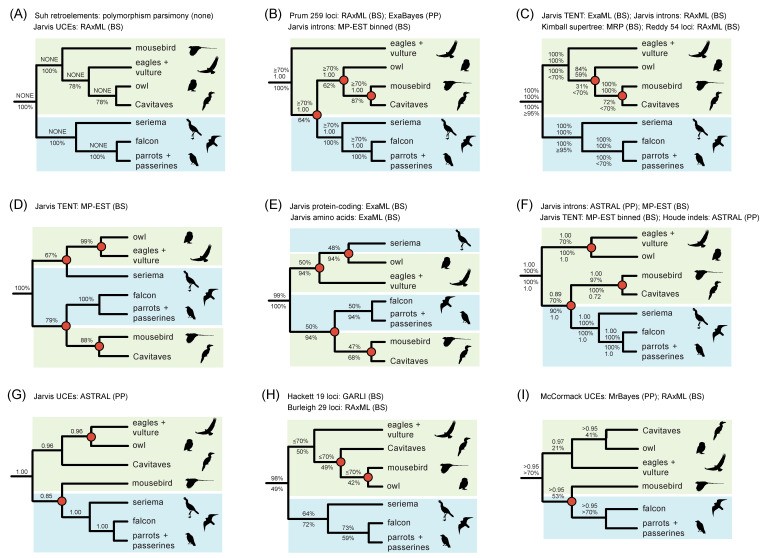
Previous phylogenetic hypotheses for Telluraves based on large molecular datasets. The RI polymorphism parsimony tree [5] is congruent with just one phylogenomic analysis, ML concatenation of UCEs (**A**), but conflicts with other published studies (**B**–**I**), many of which place Coliiformes (mousebirds) sister to Cavitaves (**B**–**F**). Clades that conflict with the RI parsimony tree are marked by red circles at nodes. Afroaves (green background) is not monophyletic relative to members of Australaves (blue background) in several topologies (**B**,**D**–**G**,**I**). Support scores are shown above and below internodes. ‘TENT’ = total evidence nucleotide tree based on protein-coding regions, introns, and UCEs. ‘Jarvis’ indicates phylogenomic datasets analyzed by the original authors [1] or subsequent reanalyses of these same datasets by other authors [7,8,43]. For Suh et al. (**A**), standard support measures were not presented [5], but counts of ILS-free IRs and conflicting IRs were given for each clade. Additional references for trees are Hackett et al. [9], McCormack et al. [10], Burleigh et al. [11], Prum et al. [2], Reddy et al. [3], Kimball et al. [12]. Silhouettes are from http://phylopic.org/ (accessed on 1 May 2020).

In addition to resolving the initial rapid radiation in Neoaves, ongoing higher-level systematic challenges include sorting out relationships within the subclade Telluraves (Afroaves + Australaves). Specifically, the monophyly of Afroaves is not secure, and interrelationships among four ancient afroavian lineages—Accipitriformes (eagles, hawks, vultures, kites), Strigiformes (owls), Coliiformes (mousebirds), and Cavitaves (cavity nesters including woodpeckers, kingfishers, hornbills, and toucans)—have been resolved in a variety of ways [1,2,3,4,5,7,8,9,10,11,12]. Nearly every possible configuration has been supported in prior phylogenomic work, often with robust support (Figure 2), and the possibility of introgressive hybridization near the base of Afroaves has been suggested based on conflicting retroelement-insertion (RI) distributions [5,6] and quartet-frequency asymmetries in sequence-based gene trees [8]. As for the inference of the overall species tree, however, gene tree-reconstruction errors may also hinder the reliable detection of introgression at deep divergences in the avian Tree of Life [4,13].

The supermatrix (concatenation) approach [14,15] does not explicitly account for the conflicting histories of different genetic loci that result from recombination, incomplete lineage sorting (ILS), and reticulate evolutionary processes. The development of summary coalescence methods [16,17,18,19,20,21] initially offered hope for accurate phylogenomic inference [22,23,24], but it quickly became apparent that coalescent methods suited for the analysis of genome-scale data are bedeviled by additional problems [13,25,26,27,28,29,30,31,32]. Most significantly, accurate reconstruction of gene trees is extremely challenging when terminal branch lengths in gene trees are long, internal branches of a species tree are extremely short, outgroups are distant, evolutionary rates vary among taxonomic groups, missing data are extensive, and the informative variation in coalescence-genes (c-genes) [33,34,35] is limited [36,37,38,39,40,41,42]. For the avian radiation, simulations that mimic the phylogenomic dataset of Jarvis et al. [1] suggest that 55–79% of internal nodes in gene trees are reconstructed inaccurately ([43]; their Table 2), which complicates reconstruction of the overall species tree. Simulation work has repeatedly demonstrated that gene tree-reconstruction errors hinder phylogenomic inference [36,38,43,44,45], a result that is also expressed in empirical systematic studies that display robust topological conflicts among species trees supported by different genomic datasets (e.g., introns vs. protein-coding regions vs. ultraconserved elements [UCEs]) and by alternative coalescent methods (e.g., MP-EST vs. ASTRAL vs. NJst vs. ASTRID), for example, [28,31,32,46].

One response to phylogenomic inaccuracy that is driven by extensive homoplasy in DNA sequence data is to collect character data that are not impacted so severely by multiple overlapping mutations, convergence, reversal, base-compositional shifts, and lineage-specific differences in rate [47,48,49]. Nucleotide sites only have a limited number of states (i.e., G, A, T, C), and with long terminal branches are expected to accrue extensive homoplasy that leads to gene tree-reconstruction error. In contrast, rare genomic changes (RGCs) such as long indels in protein-coding genes, major chromosomal rearrangements, nuclear mitochondrial (NUMT) insertions, and RIs might provide more reliable data for resolving challenging systematic problems [50,51,52]. In particular, it has been argued that RI characters may be nearly homoplasy-free systematic data [48,49,53,54] because parallel insertion at the exact same homologous genomic position by a retroelement in the same family (convergence) is thought to be extremely rare, as is the precise excision of a retroelement following insertion (reversal). Thus, conflicts in the phylogenetic distribution of RIs are generally interpreted as ‘hemiplasy’ (resulting from ILS) [55] or the products of introgression between lineages [52,56]. If RIs are reliably coded, homoplasy may be discounted as a significant complication [54], which is critical for the reliable estimation of phylogenetic relationships, branch lengths, and branch support.

For the neoavian radiation, Suh et al. [5] compiled a large matrix of 2118 RI characters for the 48 bird genomes analyzed phylogenetically in Jarvis et al. [1] and in many subsequent studies (e.g., [8]). The RI matrix provides a parallel dataset that can be directly compared to and potentially integrated with standard DNA-sequence-based datasets derived from these genomes. Suh et al. [5] executed a ‘polymorphism parsimony’ [57] analysis of the data but did not report branch lengths or traditional support values (e.g., bootstrap, decay index) for relationships in their inferred species tree. The minimum length RI parsimony tree conflicts at nearly all early branching events in Neoaves relative to summary coalescent trees based on DNA sequences [1,2,3,4,8], with relationships among major lineages of Afroaves agreeing with just one prior genome-scale analysis (Figure 2A; concatenation of UCEs) and contradicting 19 other trees (Figure 2B–I). The numerous conflicts between data types—RGCs vs. nucleotide sites—are difficult to interpret given the methodological break between the DNA-sequence-based species trees inferred using summary coalescent methods and the polymorphism parsimony analysis of RI characters. Suh [5,6] did infer distance-based networks [58] that visualized the extreme degree of conflict among RI characters and gene trees at the base of Neoaves and within Afroaves, but such networks are challenging to interpret in terms of support for competing relationships and also for disentangling conflicts due to ILS versus gene flow.

**Figure 3:**
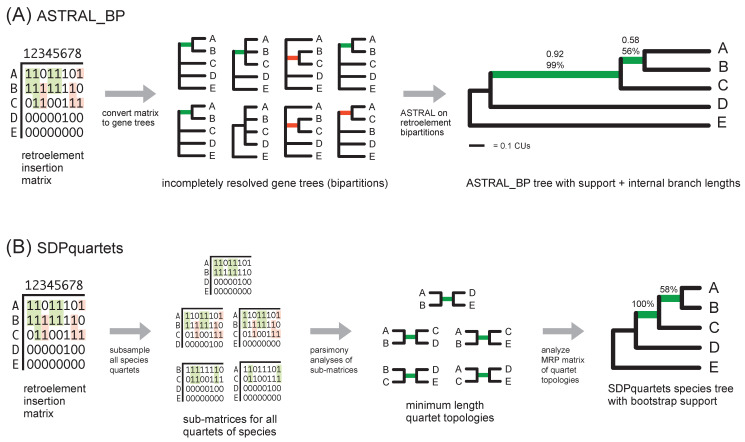
A schematic of quartet-based coalescent methods [56] that were employed for phylogenetic analyses of the avian RI matrix: (**A**) ASTRAL_BP analysis of single bipartition gene trees derived from the RI matrix, and (**B**) direct SDPquartets analysis of the RI matrix. In (**A**), internal branch lengths for the species tree are shown (terminal branches are not estimated); Bayesian local posterior probabilities and bootstrap scores are above the internal branches. In (**B**), bootstrap scores for the species tree are above the internal branches. Green internal branches in the RI bipartition gene trees (**A**) and in species quartets (**B**) are compatible with the inferred species tree, and red branches show conflicts with the species tree. Note that sample sizes (effective number of loci) for the estimation of support scores and branch lengths are small in this simple hypothetical example.

At the time when Suh et al. [5] and Suh [6] published their important work on avian RIs, ILS-aware methods that were compatible with the multispecies coalescent (MSC) were limited to RI datasets with just a few taxa (e.g., [59]). Several years later, Springer et al. [56] repurposed quartet-based coalescent methods, ASTRAL [20] and SVD-quartets [19], for the analysis of RI data (Figure 3). The newly proposed approaches for the analysis of binary RI characters, ASTRAL with bipartitions (ASTRAL_BP) and split decomposition with parsimony of quartets (SDPquartets) (Figure 3), are statistically consistent for the simulation conditions explored by Molloy et al. [60]. In ‘anomaly zone’ [61,62] simulations, where multiple short internal branches in a species tree challenge accurate phylogenetic reconstruction, ASTRAL_BP and SDPquartets outperformed various parsimony methods including polymorphism parsimony [60]. The summary coalescent methods also enable various measures of clade support/stability (Figure 3) including bootstrapping [63], local posterior probabilities (PPs) [64], partitioned coalescence support (PCS) [31] as well as internal branch length estimation in coalescent units (CUs) [60,64]. Importantly, because ASTRAL_BP is a simple extension of the general ASTRAL coalescent method to low-homoplasy binary characters (i.e., gene trees in which just a single bipartition is resolved), gene trees based on DNA or protein sequences can now be integrated into ‘total evidence’ summary coalescent analyses that also include various RGCs such as RIs, and analyses of RGCs can leverage the various statistical and descriptive outputs associated with the ASTRAL package [20,44,65,66].

In addition to hemiplasy due to ILS and rare homoplasy [67], conflicts among RI characters can also result from introgressive gene flow between hybridizing evolutionary lineages [52,59]. Several methods have been developed to quantify the asymmetrical conflicts that are expected in various hybridization scenarios. Kuritzin et al. [59] developed a statistical approach for detecting hybridization/introgression in a 3-lineage model (KKSC), while Churakov et al. [68] proposed a 4-lineage test (4-LIN) based on maximum likelihood (ML) calculations for RI datasets. In addition, Springer et al. [56] presented an unrooted quartet-based RI test for the deviation of species quartets from expectations of the MSC. This ‘quartet asymmetry test’ uses parsimony analyses on small subsets of taxa to detect potential gene-flow pathways across the overall species tree. These RGC methods have been used to infer introgressive hybridization of RIs in several clades including baleen whales and bats [56,69,70].

Here, we assessed the utility of newly proposed quartet-based summary coalescent methods for RGCs by reanalyzing a RI dataset for basal relationships of Neoaves, an extremely challenging phylogenetic problem. We used these phylogenomic analyses to address the following questions regarding the neoavian radiation and potential advantages of a quartet-based summary coalescent approach:

1) For the early radiation of Neoaves, do quartet-based coalescent analyses of the RI dataset yield congruence with a polymorphism parsimony analysis of this dataset [5] or are they in better agreement with species trees based on genome-scale coalescent analyses of DNA sequences (e.g., [1,8])?

2) For Telluraves (Afroaves + Australaves), do quartet-based coalescent analyses of RIs yield species trees that are congruent with any of the previous phylogenetic hypotheses for this clade (Figure 2)?

3) For the ASTRAL_BP tree based on RIs, do inferred branch lengths in CUs conform with the hypothesis that the initial diversification of Neoaves represents an exceptionally rapid radiation or even a ‘hard polytomy’ [6]?

4) Do quartet-based coalescent methods yield compelling support (bootstrap, local PP, stability to locus removal) for any relationships at the base of the neoavian radiation, a rapid diversification of eight or more lineages (Figure 1)?

5) Do combined ASTRAL coalescent analyses of >6000 DNA-sequence-based gene trees (introns and UCEs) with 2118 RI characters impact the species trees based on just the DNA sequence data, yielding novel insights on the neoavian radiation?

6) Does the quartet asymmetry test or alternative methods (e.g., KKSC, 4-LIN) detect evidence for gene flow among the deep lineages of Afroaves that has been hypothesized in past studies [5,6,8]?

Finally, we comment on the current utility and future prospects for using RIs to resolve challenging radiations and gene flow signals in large phylogenomic trees.

## 2. Materials and Methods

### 2.1. Datasets and Gene Trees

Suh et al. [5] published a large RI matrix for the Neoaves radiation based on genome assemblies for 48 taxa (43 ingroup taxa and five outgroups). The dataset includes 2118 RIs coded as present (1), absent (0), or missing data (?) for each taxon, with 2113 characters that are phylogenetically informative for quartet-based coalescent analyses. The data matrix was converted to 2113 single-bipartition gene trees using a previously described script (https://github.com/ekmolloy/phylotools (accessed on 17 September 2020)) from Springer et al. [56]. Each RI character was transformed to a single unrooted gene tree that includes only taxa that were coded as present or absent for that character, with a single resolved internode that separates taxa coded as present versus absent. Missing data for different species in the RI matrix are summarized in Table 1.

DNA-sequence-based gene trees derived from the same 48 avian genomes [1] as those sampled for RIs [5] were analyzed in combined coalescent analyses with the single-bipartition RI ‘gene trees’. Fully bifurcating ML gene trees for UCEs (3679 trees) and for introns (2516 trees) were from Jarvis et al. [1]. The 8251 gene trees based on protein-coding regions from Jarvis et al. [1] were not included in combined coalescent analyses because the exons from a single gene often span thousands of base pairs and are unlikely to correspond to individual c-genes [27,71,72]. Additionally, extensive homology errors have been documented in these protein-coding alignments due to various assembly, annotation, and alignment artifacts [7,73].

Combined analyses of RIs plus DNA sequences in which weakly supported gene tree nodes were collapsed [8] and/or downweighted [74] were also executed. For these differentially weighted ‘total-evidence’ [14] analyses, we utilized ML gene trees with bootstrap-support scores from [8] for UCE and intron alignments in [1]. By assigning less weight to gene tree nodes with lower bootstrap scores, the impacts of arbitrary resolution and marginal support on congruence can be assessed in a straightforward comparison of species trees with and without differential weighting.

Datasets reanalyzed here (RIs, RI bipartitions, and gene trees) are posted at: https://figshare.com/account/home#/projects/137739 (all datasets were posted 26 June 2022). Dataset S1: RI matrix from Suh et al. [5] for 48 taxa and 2118 characters. Dataset S2: 2113 RI bipartitions for ASTRAL_BP analysis. Dataset S3: 2113 RI bipartitions and 6195 fully resolved ML gene trees (introns and UCEs) [1] for the combined ASTRAL analysis with equal weights. Dataset S4: 2113 RI bipartitions and 6194 partially resolved ML gene trees [8] for the combined ASTRAL analysis with low-bootstrap nodes collapsed. Dataset S5: 2113 RI bipartitions and 6194 fully resolved ML gene trees with bootstrap supports [8] for combined weighted ASTRAL analyses.

### 2.2. Summary Coalescent Methods—Inference of Species Trees and Branch Lengths

The RI dataset [5] was analyzed using two quartet-based methods—ASTRAL_BP and SDPquartets (Figure 3). As noted above, these methods are direct extensions of ASTRAL and SVD-quartets that can be applied to RI characters or other RGCs [56,60]. Our primary analyses focused on usage of ASTRAL_BP since this versatile approach permits application of various support measures and descriptive statistics in the ASTRAL package [66] that have been applied previously to incompletely resolved gene trees (e.g., [8]). SDPquartets, an alternative quartet-based method, was used to crosscheck the optimal ASTRAL_BP topology.

For the ASTRAL_BP analyses, the set of 2113 single-bipartition gene trees derived from the RI matrix (see above) was first analyzed using ASTRAL 4.11.1 [20]. To expand the ASTRAL_BP search space, extra gene trees were input using the ‘-e’ command; we utilized 14,446 fully resolved gene trees from Jarvis et al. [1] that were based on their analyses of UCEs, introns, and protein-coding genes. A second analysis was run with ASTRAL 5.6.3 to determine whether the same optimal quartet score and topology were recovered. ASTRAL automatically outputs the topology of the species tree, the optimal quartet score, and internal branch length estimates in CUs [64]. For ASTRAL 5.6.3, the ‘-t 2′ command was used to generate additional information (e.g., quartet frequencies, local PPs for alternative resolutions, the effective number of loci [EN] for each internode). Due to missing taxa or the poor resolution of gene trees, some loci may not contribute quartets that inform the support for or against particular internodes in the optimal species tree. EN quantifies how many of the input gene trees (or RI bipartitions) contain information that bears on the quartet support/conflict at a particular internal branch.

For SDPquartets, unordered parsimony analyses were initially performed for each subset of four species in the RI matrix. Then, the optimal trees for each subset of four taxa were assembled into a species tree for the full set of species using Matrix Representation with Parsimony (MRP) [75]. Scripts in Springer et al. [56] were used to produce the MRP dataset (https://github.com/dbsloan/SDPquartets (accessed on 23 July 2020)). Parsimony analysis of the MRP dataset to search for the optimal species tree(s), those that fit the most species quartets, were heuristic using PAUP* 4.0a (build 166) with random taxon additions and tree-bisection-reconnection (TBR) branch swapping [76]. Because of the extensive missing data for passerines (five species) and outgroups (five species), which were not focal clades in the RI analysis of Suh et al. [5], numerous equally parsimonious trees were recovered (>10,000), and the initial search was truncated. To determine whether missing data in the outgroups and passerines were the primary drivers of ambiguity, a second parsimony search of the MRP dataset was executed using a ‘backbone constraint’ tree that enforced relationships for outgroups and passerines to match the well-supported species trees from Jarvis et al. [1]. This second parsimony search included 100 random-taxon-addition replicates with TBR branch swapping.

In addition to quartet-based summary coalescent analyses of the RI data, we executed an ‘equally weighted’ combined ASTRAL 5.6.3 analysis of RI bipartitions with fully resolved ML gene trees based on DNA-sequence data. This phylogenomic analysis tested whether a relatively small RGC dataset could impact tree topology and clade support (local PPs) based on more traditional molecular data in a ‘total evidence’ coalescent analysis of both data types. The resulting combined-data species tree was also used to detect emergent RI support for any contentious clades that were not supported by separate ASTRAL_BP analysis of the RI dataset (see Section 2.4 below).

Three additional combined analyses of RIs and gene trees (UCEs + introns) tested whether the downweighting of marginally supported gene tree nodes drives any strongly supported conflicts between our species tree based on RIs versus species trees based on RIs + DNA sequences. Weakly supported nodes are common in DNA-sequence-based gene trees (e.g., [25,29,30]). Such nodes were either collapsed or severely downweighted relative to the RI bipartitions. In the first analysis, gene tree nodes with ≤5% bootstrap support were collapsed [8], and a combined ASTRAL 5.6.3 search of the incompletely resolved gene trees and RIs was executed. In the second analysis, gene tree nodes were differentially weighted according to the level of bootstrap support (from 0–100), and the maximum weight (100) was applied to each RI bipartition. This weighting scheme assumes that RIs are extremely reliable phylogenetic characters [47,48,49], but no more reliable than a gene tree node with 100% bootstrap support. In the final combined analysis, gene tree nodes with bootstrap support from 0 to 49% were collapsed, nodes with bootstrap support ≥50% were differentially weighted from 50–100, and RIs were again weighted 100. The last two combined-data searches were executed using a recently developed ‘weighted-ASTRAL’ (wASTRAL) approach [74] with the Accurate Species Tree EstimatoR (ASTER*) software that is publicly available at https://github.com/chaoszhang/ASTER (accessed on 21 May 2022).

Species trees supported by our various ASTRAL analyses of Datasets S2–S5 are posted at: https://figshare.com/account/home#/projects/137739 (accessed on 26 June 2022). All species trees are in ‘Tree file S1’.

### 2.3. Summary Coalescent Methods—Clade Support and Stability Indices

Multiple support indices were compared for the ASTRAL_BP tree based on RI data. Bayesian local PPs [64] are automatically output by ASTRAL for all internal branches. ASTRAL_BP bootstrapping (100 pseudoreplicates) of 2113 RI bipartitions was executed using ASTRAL 5.6.3 with the ‘-e’ command to broaden the tree search using 14,446 fully-resolved gene trees from Jarvis et al. [1]. Bootstrap percentages were mapped onto the optimal ASTRAL_BP tree. In addition to local PPs and bootstrapping, we assessed the stability of nodes supported by ASTRAL_BP analysis by estimating the minimum number of RI-bipartition removals required to collapse a particular node, a ‘locus removal index’. We implemented the approach outlined in Gatesy et al. [31]. First, ‘anti-constraints’ were incorporated into ASTRAL_BP searches of the RI dataset to find the best scoring topology that lacked the node of interest [77]. Thirty-four anti-constraint searches were run using ASTRAL 5.6.3 with the ‘--remove-bipartitions’ command. Each anti-constraint analysis yielded a fully resolved species tree that contradicted the node of interest. By subtracting the quartet score for the anti-constraint tree from the quartet score for the optimal ASTRAL_BP tree (=‘coalescence support’) and then partitioning the quartet support among the 2113 RIs (=‘partitioned coalescence support’; PCS), the number of retroelement removals required to collapse a node can be estimated. We used the methods and scripts (https://github.com/dbsloan (accessed on 15 May 2020)) from Gatesy et al. [31] to calculate PCS scores at each node of interest, to rank the RI loci according to quartet support at a given node, and to estimate the minimum number of RI removals required to collapse each node. Additional ASTRAL_BP searches in which multiple RI characters were removed from analysis were run to test whether the supports at multiple nodes in the optimal ASTRAL_BP tree were interdependent.

### 2.4. Hidden (Emergent) RI Support

For summary coalescent methods, analyses with both contrived and simulated datasets demonstrate that it is possible to produce species trees with clades that conflict with all of the input gene trees that were used to generate the species tree [25,78]. Analogous ‘hidden support’ has been quantified in parsimony and ML concatenation analyses that include multiple data partitions [79,80]. Given the very short internal branches at the base of Neoaves, we hypothesized that coalescent support for some clades might be completely emergent (i.e., not supported cleanly by any RI bipartitions). For the 2113 RI dataset and for the combined dataset of 2113 RIs plus 6195 ML gene trees (UCEs and introns with ‘equal weighting’), RI characters were mapped by parsimony (unordered) onto species trees using PAUP*. Unequivocally optimized synapomorphies that change just once on the tree (in this parsimony context) were recorded at contentious nodes to determine whether any RI characters cleanly support a given clade (or not). When there were no such changes at an internode in the coalescent tree based on RIs, the clade was considered ‘emergent’. We also noted uniquely derived, ‘hidden synapomorphies’ [79] in the RI dataset that emerged in the combined ASTRAL analysis of RIs + introns + UCEs but were not synapomorphic in the ASTRAL_BP tree based on just RIs. Note that for convenience, we refer to uniquely derived, unequivocally optimized synapomorphic characters in the remainder of the paper as ‘perfectly congruent RI synapomorphies’ due to the fact that these characters can be explained by a single insertion with no evidence for conflicting homoplasy, ILS, or gene-flow patterns.

### 2.5. Inference of Introgressive Hybridization and Phylogenetic Networks

The possibility of introgressive hybridization within Afroaves [5,6,8] was explored via the quartet asymmetry test for RI data [56], the KKSC test [59], a newly developed 4-LIN test for RCGs [68], and related statistics that measure asymmetrical support for suboptimal resolutions at an internal node in the species tree. Asymmetries between minority resolutions at controversial nodes within Afroaves were first characterized using the full RI dataset (48 taxa, 2113 characters) and the optimal ASTRAL_BP tree for this dataset. The following were tabulated at controversial nodes and for majority and minority resolutions: (1) counts of perfectly congruent RI synapomorphies; (2) local Bayesian PPs; (3) PCS scores; and (4) quartet frequencies.

The quartet asymmetry test [56] examines the differential RI character support for the majority resolution supported by ASTRAL_BP and for the two minority resolutions for a given quartet of species. Under the MSC, the two minority resolutions of a particular species quartet should have nearly equal support (within sampling error), and strong deviations from symmetry imply gene flow or other incompatibilities with the MSC. The quartet asymmetry test was applied to exemplars from four major lineages of Afroaves (Coliiformes, Strigiformes, Cavitaves, Accipitriformes) and a close outgroup (Australaves) by sampling species with the least missing data for the RI matrix (*Colius striatus* [mousebird], *Tyto alba* [owl], *Leptosomus discolor* [cuckoo-roller], *Haliaeetus leucocephalus* [eagle 2], and *Cariama cristata* [seriema]) (Table 1). All possible species-quartets for this subset of five taxa were examined. The significance of asymmetry was tested using a binomial distribution (two tailed) with an expectation of equal probability (0.5) for either of the two suboptimal resolutions at a supported node using an online tool (https://www.socscistatistics.com/tests/binomial/default2.aspx (accessed on 4 May 2020)).

Given the prior evidence for nearly equal RI support for two conflicting resolutions of a trichotomy among Strigiformes, Cavitaves, and Accipitriformes [1,5], additional statistical tests were used to explore the possibility of introgression/hybridization within Afroaves. Using the RI dataset [5], KKSC statistics [59] were applied to three character-state patterns (011, 101, 110) for three species (*C. striatus*, *T. alba*, *L. discolor*). An online application (http://retrogenomics.uni-muenster.de:3838/KKSC_significance_test/ (accessed on 10 June 2022)) was employed to assess the relative support for seven topologies including three networks that imply introgressive hybridization. The 4-LIN test [68] is an extension of the 3-lineage KKSC to four species. 4-LIN uses log-likelihood ratio tests to compare 155 different topologies, 129 of which incorporate different hybridization/introgression scenarios. The quartet-asymmetry test, which is also applied to four species, compares the RI support for just three different patterns (0011 + 1100, 0101 + 1010, 0110 + 1001), while the 4-LIN test utilizes RI data for ten different patterns (0111, 0011, 0101, 0110, 1011, 1001, 1010, 1101, 1100, 1110). Missing data are not permitted in any character for the KKSC and 4-LIN tests. To reduce the impact of taxa with extensive missing data, the 4-LIN test was performed on two datasets: (1) the same four afroavian species that were utilized in the quartet asymmetry test (*C. striatus*, *T. alba*, *L. discolor*, and *H. leucocephalus*), and (2) three species of Afroaves (*C. striatus*, *T. alba*, *L. discolor*) and an outgroup species (*C. cristata*). A web R-application (http://retrogenomics.uni-muenster.de:3838/hammlet/ (accessed on 14 May 2022)) was utilized to perform 4-LIN tests and to assess the significance of support for introgressive hybridization (KKSC on subsets of three species).

## 3. Results

### 3.1. Species Trees and Branch Lengths

**Figure 4:**
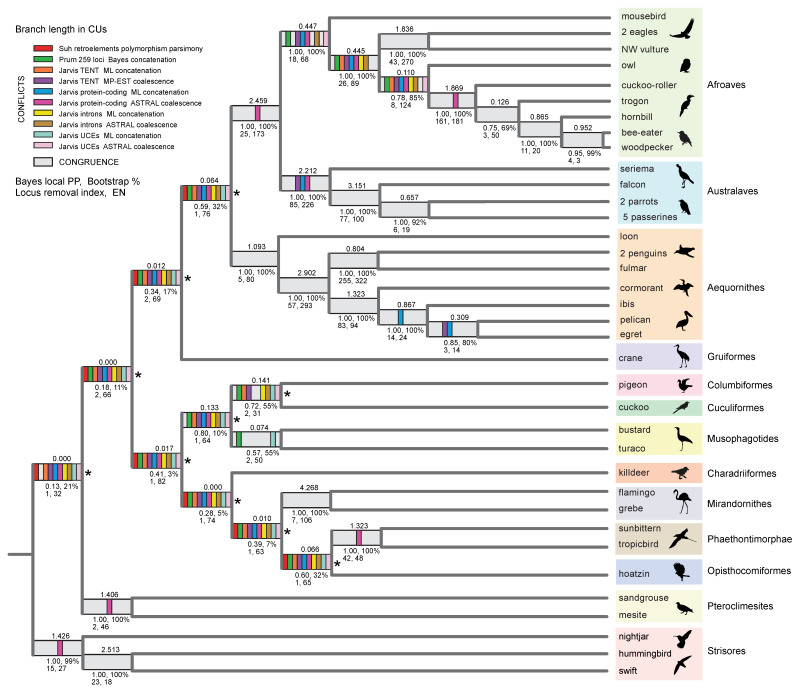
Species tree for Neoaves based on the ASTRAL_BP and SDPquartets coalescent analyses of the RI dataset. Both quartet-based summary coalescent methods support the same optimal topology for the ingroup. ASTRAL_BP branch lengths in coalescent units (CUs) are above the internodes. Various support scores (Bayesian local posterior probability [PP], ASTRAL_BP bootstrap, ASTRAL_BP locus removal index), and effective number of loci (EN) are below the internodes. Topological conflicts with ten earlier genome-scale studies are marked by colored bars, while congruence with these studies is shown in light gray. Ten deep nodes that show wholesale conflicts with previous studies are marked by asterisks (*). ‘Locus removal index’ is the minimum number of RI-locus removals required to collapse a node in ASTRAL_BP analysis. Outgroup taxa to Neoaves (chicken, turkey, duck, tinamou, ostrich) are not shown. ‘TENT’ = total evidence nucleotide tree based on protein-coding regions, introns, and UCEs. ‘Jarvis’ indicates phylogenomic datasets analyzed by the original authors [1] and subsequent reanalyses of these same datasets by other authors [7,8]. Additional references are Suh et al. [5] and Prum et al. [2]. Silhouettes are from http://phylopic.org/ (accessed on 1 May 2020).

ASTRAL_BP and SDPquartets analyses of the RI dataset supported the same basic tree topology (Figure 4). Relationships among the five outgroup taxa and among the five passerines were unresolved in the large set of >10,000 optimal trees saved in the SDPquartets analysis (minimum treelength = 1,336,560 steps for the MRP matrix of 1,167,480 species-quartets). This was due to a lack of informative character variation among the outgroup species and among passerines. A parsimony search of the MRP matrix guided by a ‘backbone constraint’ that specified outgroup and passerine relationships as in the sequence-based phylogenomic trees of Jarvis et al. [1] yielded a single SDPquartets topology with the same treelength and relationships as in Figure 4.

**Figure 5:**
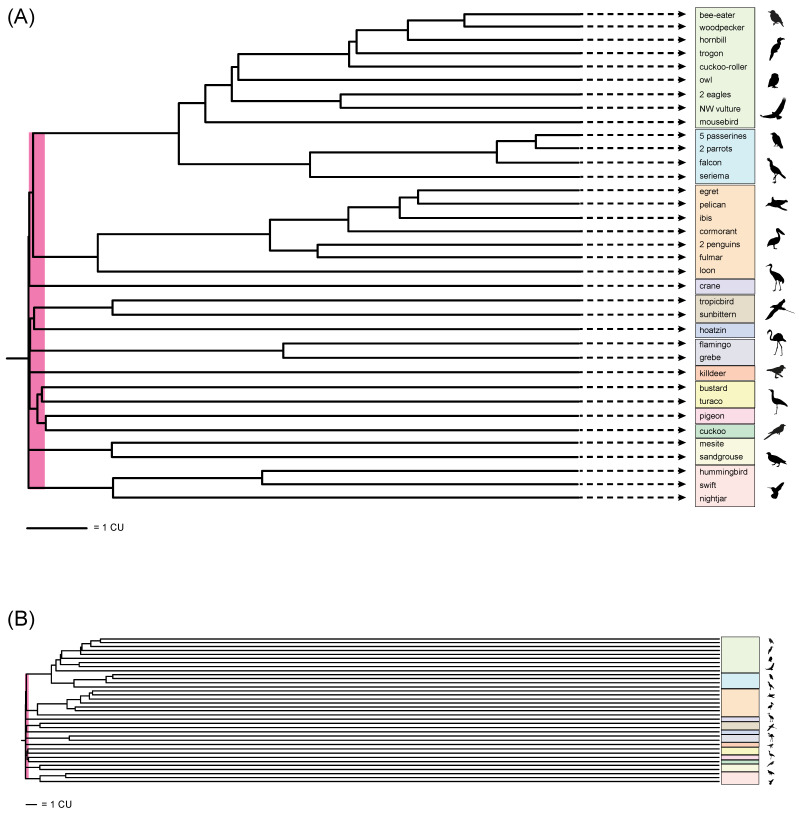
ASTRAL_BP phylogram for Neoaves based on RIs with branch lengths in coalescent units (CUs) shown at two scales. In (**A**), a focus on the base of the phylogram shows the very short branch lengths near the initial diversification of Neoaves (pink bar represents 0.25 CUs); terminal branches are truncated (indicated by dashed lines and arrowheads). In (**B**), estimates of terminal branch lengths are based on conversion of time in millions of years (MY) from a molecular clock estimate [1] to coalescent units (CUs). A conversion of one MY to one CU was used [27,56]. Note the extreme lengths of terminal branches relative to the extremely short branches at the base of Neoaves (pink bar). Silhouettes are from http://phylopic.org/ (accessed on 1 May 2020).

For ASTRAL, only a single optimal topology is saved per tree search. Multiple ASTRAL_BP analyses with different random starting points and using two versions of ASTRAL (v. 4.11.1, v. 5.6.3) yielded optimal species trees (quartet score = 12,235,132) that were generally congruent with each other and with the SDPquartets results (Figure 4). Conflicts among the optimal species trees from independent searches were again restricted to relationships among the outgroups and among passerines, for which Suh et al. [5] did not code informative RIs given the focus of their study. For the ASTRAL_BP tree, estimated internal branch lengths in CUs are shown numerically in Figure 4 and for the corresponding ASTRAL_BP phylogram in Figure 5. Because terminal branches are not estimated by ASTRAL (branch lengths are based on conflicts among quartets/loci), terminal branch lengths were approximated by converting molecular-clock divergences ([1]; their Figure 1) to CUs using gross estimates of average population size and generation time for bird species. A conversion of one million years (MY) to one CU was implemented [27,56].

### 3.2. Clade Congruence, Support, and Stability

**Figure 6:**
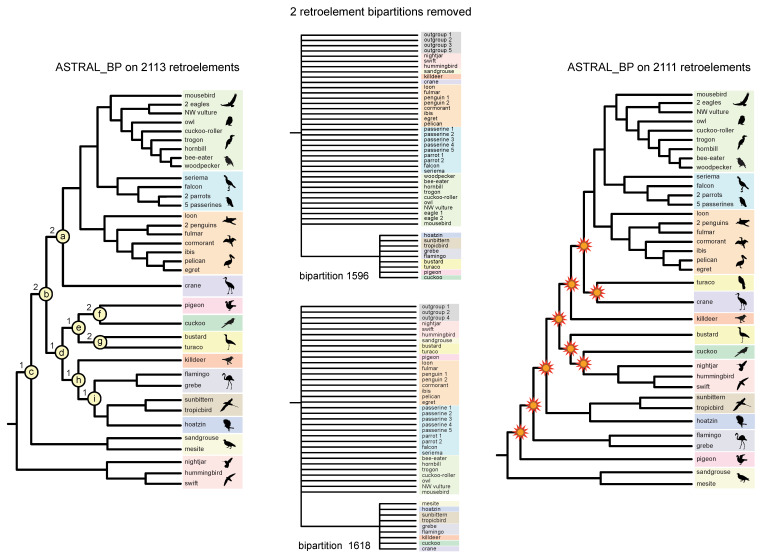
Interdependent “house of cards” support at the base of Neoaves for ASTRAL_BP coalescent analysis of RI bipartitions. ASTRAL_BP analysis of all 2113 RI bipartitions supports the optimal species tree and the controversial ‘clades a–i’ (**left**), but removal of just two RI bipartitions (#1596 and #1618) (**middle**) from the analysis results in a species tree (**right**) in which nine clades conflict with the ASTRAL_BP tree supported by all 2113 bipartitions (starbursts at nodes). Note that the two critical RIs that determine support for ‘nodes a–i’ conflict with the optimal ASTRAL_BP tree based on the full dataset (**left**). Silhouettes are from http://phylopic.org/ (accessed on 1 May 2020).

**Figure 7:**
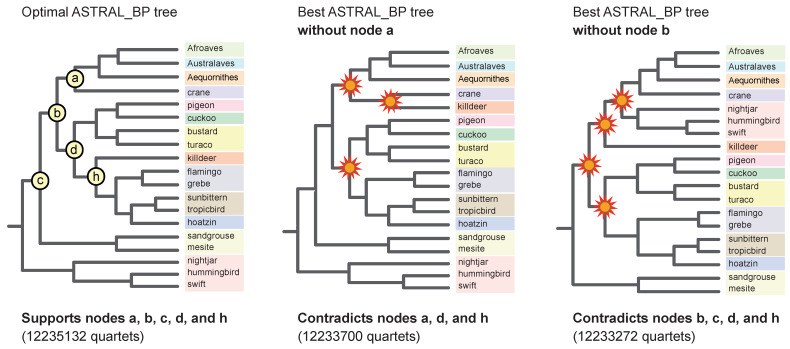
Examples of interdependent ‘linked’ support at multiple nodes in the ASTRAL_BP tree for RI characters. The optimal ASTRAL_BP tree (**left**), the best ASTRAL_BP tree that lacks ‘clade a’ (**middle**), and the best ASTRAL_BP tree that lacks ‘clade b’ (**right**) are shown. In each case, the best species tree found in an ‘anti-constraint’ ASTRAL_BP search [77] lacked the clade of interest but also conflicted with several other clades in the optimal ASTRAL_BP tree. Support for multiple nodes is interdependent (‘linked’) [81]. Clades in the suboptimal trees that conflict with the best ASTRAL_BP tree are marked by starbursts. Quartet scores are shown for each tree. Relationships among taxa within Afroaves, Australaves, and Aequornithes are not shown but exactly match the optimal ASTRAL_BP tree (Figure 4).

ASTRAL_BP support scores (Bayesian local PPs, bootstrap percentages), stability to removal of RI bipartitions, EN, and congruence (light gray) versus conflict (colored bars) with ten prior genome-scale trees are shown for the quartet-based coalescent species tree in Figure 4. Examples that further demonstrate instability to the removal of RI bipartitions and interdependent support across multiple internal nodes (‘linked branch support’) [81] are shown in Figure 6 and Figure 7.

### 3.3. Hidden (Emergent) RI Support and Combined (‘Total Evidence’) Analyses

**Figure 8:**
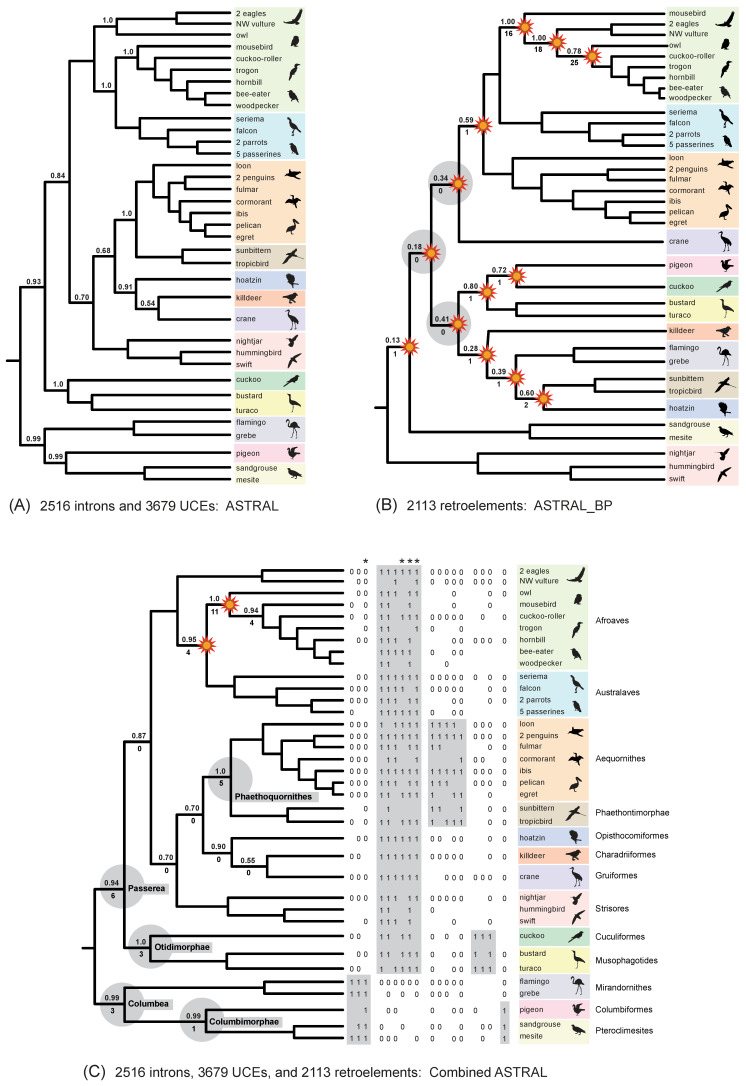
Separate and combined coalescent analyses of RIs revealed emergent RI support for controversial clades. ASTRAL species trees for three datasets are shown: (**A**) gene trees for 2516 introns and 3679 UCEs [1]; (**B**) 2113 RI bipartitions [5]; (**C**) 6195 sequence-based gene trees plus 2113 RI bipartitions with equal weighting. Local posterior probabilities (PPs) are above internodes that conflict among the three trees. For analyses that included RIs, the number of perfectly congruent RI synapomorphies is below each internode. In (**B**) and (**C**), clades that are incongruent with the species tree for introns + UCEs are marked by starbursts. In (**B**), there are three completely emergent clades that are not unequivocally supported by even a single perfectly congruent RI (gray circles). In (**C**), 18 ‘hidden’ RI synapomorphies that emerge in combined analysis support five basal clades of Neoaves (gray) that conflict with the separate ASTRAL_BP analysis of RIs. For each of these 18 characters (columns to the right of taxa), retroelement present = 1, retroelement absent = 0, missing data = no code. Asterisks (*) mark four RI characters that were coded as missing data in outgroups but map to deep nodes in Neoaves if retention of ancestral polymorphism is minimized. Silhouettes are from http://phylopic.org/ (accessed on 1 May 2020).

**Figure 9:**
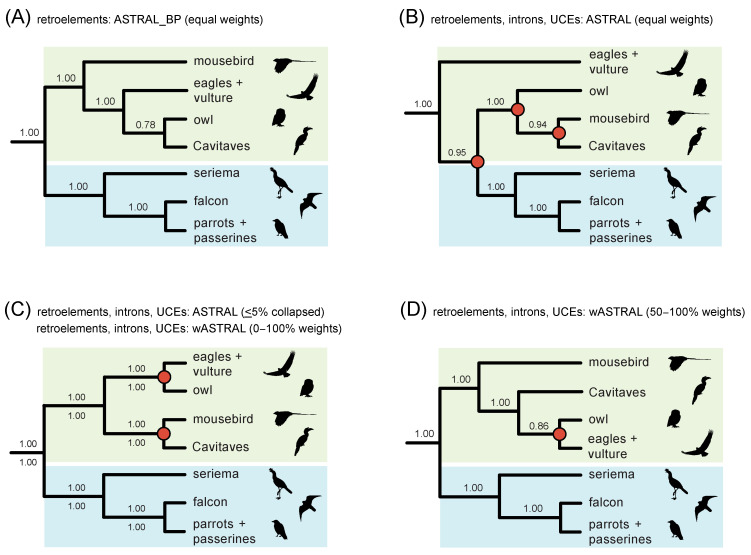
Within Telluraves, downweighting of poorly supported gene tree nodes [8,74] improves congruence to the RI species tree in combined phylogenetic analyses of RIs and sequence-based gene trees. (**A**) ASTRAL_BP on RI bipartitions; (**B**) combined ASTRAL with equal weighting of RIs and fully resolved gene trees; (**C**) combined ASTRAL on RI bipartitions and gene trees with nodes ≤5% bootstrap collapsed; combined wASTRAL with RI bipartitions weighted 100 and gene tree nodes weighted from 0–100 based on bootstrap percentages; (**D**) combined wASTRAL with RI bipartitions weighted 100 and gene tree nodes weighted from 50–100 (nodes with 0–49% bootstrap collapsed). Local PPs are at internodes, and clades that conflict with the ASTRAL_BP analysis of RIs (**A**) are marked by red circles (**B**–**D**).

In our phylogenetic results, hidden RI support for phylogenetic relationships within Neoaves was expressed in two ways (Figure 8). First, three clades in the optimal ASTRAL_BP species tree were not resolved in any of the input 2113 RI bipartitions. These three clades have no perfectly congruent RI synapomorphies and are therefore completely ‘emergent’ clades that are only supported by conflicting RI bipartitions (Figure 8B). Second, we recorded perfectly congruent RI synapomorphies that provided emergent support for clades in the combined-data ASTRAL tree based on RIs, introns, and UCEs (Figure 8C). Eighteen ‘hidden synapomorphies’ that provide unequivocally optimized support in this equally weighted ‘total evidence’ species tree corroborate five controversial higher-level clades within Neoaves (Figure 1) that were not supported by separate coalescent analysis of the RI data (Figure 8B). Additionally, two unique clades within Telluraves were supported by the combined ASTRAL tree (Figure 8C) that conflict with a separate analysis of the DNA-sequence-based gene trees (Figure 8A) and with separate analysis of the RIs (Figure 8B). This indicates that even comparatively small RI datasets can overturn clades that are robustly supported (local PP = 1.0) by thousands of gene trees (Figure 8A) in combined summary coalescent analyses (Figure 8C). Results for three additional combined ASTRAL searches in which weakly supported nodes in gene trees were collapsed and/or downweighted are shown in Figure 9.

### 3.4. Evidence for Introgressive Hybridization

**Figure 10:**
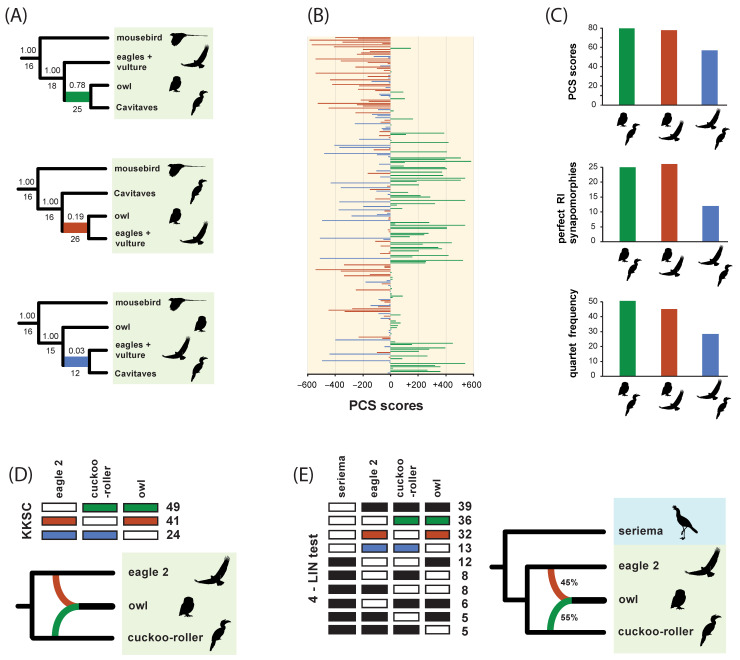
Conflicting RI evidence for deep relationships within Afroaves. (**A**) The ASTRAL_BP coalescent analysis of RIs supports Strigiformes (owl) as sister to Cavitaves; local branch swaps relative to the optimal hypothesis are also shown with Bayesian local PPs above internodes and perfectly congruent RI synapomorphies below internodes. (**B**) Positive partitioned coalescence support (PCS) scores for the optimal tree (green) and negative PCS scores for the two alternative resolutions (red and blue) are shown. (**C**) Asymmetrical RI support for three alternative placements of owl is shown for PCS scores, perfectly congruent RIs, and quartet frequencies. (**D**) Counts for three site patterns (green, red, blue = retroelement present, white = retroelement absent) used to reconstruct a KKSC [59] network for the three species that are shown. (**E**) Counts for ten site patterns (black, green, red, blue = retroelement present, white = retroelement absent) used to reconstruct a 4-LIN [68] network for four species. Silhouettes are from http://phylopic.org/ (accessed on 1 May 2020).

Asymmetrical RI support for minority resolutions at the controversial Strigiformes + Cavitaves node within Afroaves was detected using a variety of methods (Figure 10). For the full RI dataset, related indices (Bayesian local PPs, perfectly congruent RI synapomorphies, PCS scores, quartet frequencies) measured an excess of support for Strigiformes + Accipitriformes relative to Cavitaves + Accipitriformes (Figure 10A–C). For a subsample of five taxa (four members of Afroaves and an outgroup), the quartet asymmetry test did not identify significant asymmetry for any species-quartets. The KKSC and 4-LIN approaches (Figure 10D,E) inferred networks with *T. alba* (owl; Strigiformes) interpreted as a hybrid taxon with genetic contributions from *L. discolor* (cuckoo-roller; Cavitaves) and *H. leucocephalus* (eagle 2; Accipitriformes). For KKSC, a network was supported (p = 0.046) relative to bifurcating trees or a polytomy among owl, eagle 2, and cuckoo roller (Figure 10D). For one set of four species (owl, eagle 2, cuckoo-roller, seriema), a 4-LIN network was supported relative to 154 alternative phylogenetic hypotheses that included fully bifurcating trees, trees with polytomies, and networks with one or more gene flow pathways. For RI characters included in this 4-LIN test (Figure 10E), asymmetry was more extreme, and KKSC was again significant for owl, eagle 2, and cuckoo-roller (*p* = 0.014). The 4-LIN test for a second set of four species (owl, eagle 2, cuckoo-roller, mousebird) yielded ambiguous results. When run multiple times (‘stepwise empirical’ and ‘reverse empirical’ options), the online application (http://retrogenomics.uni-muenster.de:3838/hammlet/ (accessed on 14 May 2022)) alternatively output a bifurcating tree that was congruent with our ASTRAL_BP tree (Figure 4) or a tree in which mousebird was sister to a clade of three species (owl, eagle 2, cuckoo-roller) that form a trichotomy.

## 4. Discussion

### 4.1. Quartet-Based RI Species Trees

At deep nodes in the neoavian radiation, quartet-based coalescent analyses of the RI matrix did not yield high congruence with a prior polymorphism parsimony analysis of this same dataset [5] nor to any species-tree hypotheses for Neoaves based on genome-scale coalescent analyses of DNA-sequence gene trees (e.g., [1,2,8]). ASTRAL_BP and SDPquartets analyses of the RI dataset infer the same branching sequence at these ancient nodes despite the very different ways that these methods utilize quartets to infer species–tree hypotheses (Figure 3). However, the resulting quartet-based species tree showed nearly complete incongruence with ten prior genome-scale analyses (both concatenation and coalescent) at ten deep nodes (Figure 4). For these early branching events, only two of the ten clades were congruent between coalescent analyses of RIs and polymorphism parsimony analysis of these same data (pigeon + cuckoo; pigeon + cuckoo + bustard + turaco), and one deep clade (Phaethoquornithes) that was not supported by ASTRAL_BP was resolved in several sequence-based trees and by the polymorphism parsimony analysis of RIs [5]. The sister-group relationship between Strisores (nightjar + hummingbird + swift) and all remaining neoavian species recovered in the RI-based coalescent tree (Figure 4) has been corroborated by a few studies. Most prominently, ML and Bayesian concatenation analyses of a supermatrix for 198 species and 259 nuclear loci [2] supported this surprising relationship (local PP 1.0 and 61% ML bootstrap for all Neoaves except Strisores). An early divergence of Strisores in Neaoves was also resolved by a subset of analyses in Jarvis et al. [1] that were based on protein-coding sequences from 8251 genes. Support for a clade including all Neoaves except Strisores was found for low variance exons (the 10% with the least base-compositional heterogeneity; 52% ML bootstrap), 1st + 2nd codon positions (96% ML bootstrap), 1st codon positions (55% ML bootstrap), and amino-acid sequences (61% ML bootstrap). More recently, MP-EST coalescent analysis of 6931 conserved nonexonic elements (CNEEs) also supported an initial split in Neoaves between Strisores (100% bootstrap) and all remaining species in Neoaves (86% bootstrap), but taxonomic sampling was not as complete in this study (20 species within Neoaves), because the primary focus was on palaeognath relationships [82].

Reddy et al. [3] interpreted the very early divergence of Strisores as an artifact driven by protein-coding loci that are prone to base-compositional shifts and other complexities in sequence evolution that fit poorly to the relatively simple substitution models commonly utilized in genome-scale ML analyses. It is therefore intriguing that the summary coalescent tree for RIs supports a sister-group relationship between Strisores and the remaining species of Neoaves, because RIs do not share the same substitution biases that afflict protein-coding DNA sequences and amino acid sequences. Overall, however, relationships at the base of Neoaves in the quartet-based coalescent tree for RIs (Figure 4) consistently clash with prior phylogenomic work at nearly every early divergence. This is the same region of the overall tree that has vexed prior attempts at resolving the early radiation of Neoaves using genome-scale data, wherein disagreements between different genomic datasets and among alternative tree-building methods are common and often robustly supported in alternative configurations [3,4].

In addition to nearly universal incongruence with previous phylogenomic work at the earliest branching events in Neoaves (Figure 4), the quartet-based species tree for RI characters also conflicts with most prior genome-scale hypotheses for Telluraves, an ecologically diverse clade of core landbirds. Among DNA-sequence based trees, just a single ML concatenation analysis of UCEs [1] was wholly congruent for the major lineages of Telluraves shown in Figure 2, with other hypotheses conflicting at various nodes, often with high support (Figure 2B–I). Most prior trees position Coliiformes (mousebird) sister to Cavitaves, commonly with maximal support (Figure 2B–F), and Afroaves is not even monophyletic in many published trees (Figure 2D–G, I). The position of Strigiformes (owl) is scattered across published trees, alternatively sister to Cavitaves (Figure 2A,I), Coliiformes + Cavitaves (Figure 2B,C), Accipitriformes (Figure 2D,F,G), Cariamiformes (Figure 2E), or Coliiformes (Figure 2H). Despite this jumble of phylogenetic hypotheses (Figure 2), our quartet-based species tree is completely congruent with the earlier polymorphism parsimony analysis of RIs for relationships in Telluraves (Figure 2A). Both RI trees place Strigiformes as sister to Cavitaves (cuckoo-roller, trogon, hornbill, bee-eater, woodpecker), with Accipitriformes (eagles, NW vulture) and Coliiformes (mousebird) branching from successively deeper nodes within a monophyletic Afroaves (Figure 4).

### 4.2. Quartet-Based RI Branch Lengths in Coalescent Units (CUs)

Inferred branch lengths in CUs for the quartet-based RI species tree generally support the hypothesis that the initial diversification of Neoaves represents an exceptionally rapid radiation or perhaps even a ‘hard polytomy’ [6]. Indeed among the ten highly conflicting nodes at the base of Neoaves, three internal branch lengths are 0.000 CUs, and another five internal branches are less than 0.070 CUs (Figure 4). For context, only two adjacent internal branches that are each 0.1568 CUs in length can generate an ‘anomaly zone’ in which the most probable gene tree is expected to be incongruent with the species tree [83]. In our ASTRAL_BP species tree, 11 internal branches that were <0.157 CUs were clustered near the base of Neoaves (Figure 4 and Figure 5). For short branch lengths such as these (<0.300 CUs), Molloy et al. [60] suggested that ASTRAL_BP on RI data, if accurately coded and assuming no homoplasy with a constant accumulation rate, provide reliable branch lengths. In contrast, extensive gene tree reconstruction errors in standard sequence-based analyses strongly bias estimates of species–tree branches toward shorter lengths due to extensive homoplasy at nucleotide sites, insufficient informative variation, poor fit to overly simple nucleotide substitution models, long-branch misplacement, and other problems. This effect is expected to be especially strong for rapid radiations that are deep in time such as the initial diversification of Neoaves [38,43], resulting in stunted ‘bonsai’ species trees [25,26,27], particularly if internodes that are arbitrarily resolved are not accounted for [8,66,84,85]. In contrast, because RIs are commonly interpreted as low-homoplasy events, branch lengths based on these RGCs are an important ‘reality check’ on branch-length estimation based on conflicts among sequence-based gene trees at a particular node.

The remarkably short ASTRAL branch lengths inferred from conflicts among low-homoplasy RIs, in particular three branches estimated at 0.000 CUs, provide additional corroborating evidence for the hypothesis that the initial diversification of Neoaves was a starburst of speciation, or nearly so [6]. Assuming a general conversion of one CU to one MY [27,56], 11 or more extant bird lineages diversified within a relatively short interval of time that spans ~250,000 years (Figure 5B). Patel et al. [38] suggested a rough conversion of 400,000 years to one CU for a typical bird species, which would compress the initial rapid diversification of Neoaves from ~250,000 years to just ~100,000 years, and further increase the disparity between very short internal branch lengths at the base of Neoaves and the much longer terminal branches that extend from the Cretaceous–Paleogene boundary to the present (Figure 5B). The RI-based branch lengths suggest that the early radiation of Neoaves represents a daunting systematic challenge, and the very low support scores in our ASTRAL_BP tree might better represent the extremely tight branching sequence in this sector of the avian tree (Figure 4) relative to prior sequence-based phylogenomic trees that conflict strongly with each other at these same nodes [1,2,3,5,7,8,9,10,11,12,43,82]. Many more low-homoplasy RGCs presumably would be required to solve this monumental phylogenetic puzzle.

In contrast, for relationships within Telluraves, a second sector of the overall tree for Neoaves where phylogenomic resolution has been highly contentious (Figure 2), no internal branch lengths were shorter than 0.070 CUs (Figure 4). The ASTRAL_BP phylogram clearly expresses the difference in scale for the Telluraves diversification versus the initial radiation of Neoaves (also see related discussion in [5,6]). Although still deep in time, the divergences within Telluraves are at least visible in the overall phylogram, assuming a one CU to one MY conversion (Figure 5). The internal branch that subtends the Strigiformes + Cavitaves clade is the shortest within Telluraves (0.110 CUs), perhaps explaining the difficulties in placing owls relative to other major lineages of Afroaves in previous work based on genome-scale analyses of DNA sequences. However, given that gene flow between lineages has been hypothesized in this region of the tree [5,6,8], ASTRAL_BP branch lengths might be distorted because introgression is not accounted for in this quartet-based coalescent method.

### 4.3. Clade Support and Stability: ‘House-of-Cards’ RI Support at the Base of Neoaves

Quartet-based coalescent methods do not yield compelling support (bootstrap, local PP, stability to locus removal) for any relationships at the base of the neoavian radiation, a rapid diversification of 11 or more lineages (Figure 4). It should be noted that for all of the deep nodes in the RI tree that show wholesale conflicts with previous genome-scale studies, there is no overwhelming consensus among prior phylogenomic work for alternative resolutions of these nodes. Various studies, instead, have presented conflicting alternative resolutions, often with high bootstrap or local PP support scores (e.g., [1,2,8]). In contrast, all deep divergences in Neoaves that are delimited by very short internal branches in the ASTRAL_BP tree (Figure 5) also have extremely low standard support scores. For the ten high-conflict clades that are marked by asterisks in Figure 4, Bayesian local PPs range from just 0.13 to 0.80, and bootstrap scores range from just 3 to 32%. Internal branches in the overall ASTRAL_BP species tree that are much longer (in CUs) generally have high Bayesian local PPs, bootstrap supports, and congruence with previous phylogenomic analyses (Figure 4). For relationships in Telluraves, where estimated branch lengths are relatively long, nine clades had high Bayesian local PPs (0.95–1.0) and bootstrap support scores (92—100%). One exception is the Strigiformes + Cavitaves clade with a relatively short subtending branch (0.110 CUs) and less impressive support (0.78 PP, 85% bootstrap).

Support for a particular clade also has been expressed as stability to the removal of data—characters, genes, datasets, or taxa [79,86,87,88,89,90,91]. Here, we further assessed the stability of clades by estimating the minimum number of RI-bipartition removals that are required to collapse a clade in ASTRAL_BP analysis, a ‘locus-removal index’ that is analogous to the ‘clade stability index’ of Davis [87]. In these calculations, a constrained ASTRAL_BP search and PCS were used to find the most influential RI bipartitions at a given supported node [31,56]. For the ten very short, highly conflicting branches near the base of Neoaves, only one or two RI-bipartition removals were enough to collapse each of these unstable clades (Figure 4). Relationships within Telluraves are generally much more stable to the removal of RI bipartitions. For example, very high locus-removal indices were estimated for Telluraves (25 loci), Australaves (85 loci), and Afroaves (18 loci). The overwhelming RI support for the monophyly of Australaves is in contrast to several genome-scale analyses that do not even resolve this clade (Figure 2D,E). For the controversial Strigiformes + Cavitaves clade within Afroaves, stability to RI removal was more modest (8 loci), consistent with the moderate support at this node according to standard measures (Figure 4).

To calculate the locus removal index, the PCS scores for RI loci are ranked from highest to lowest at each node, and this output can reveal potentially interrelated (interdependent) support at multiple nodes. For example, several RI bipartitions (e.g., #s 69, 1596, and 1618 in the set of 2113 informative RIs) showed high influence, according to PCS, at multiple early divergences in the ASTRAL_BP tree. Removal of various combinations of these highly influential RI loci shows that support at the base of the ASTRAL_BP tree is like a ‘house of cards’, consistent with the very short branch lengths (Figure 5) and very low traditional support scores (Figure 4) at the initial, rapid radiation of Neoaves. Removal of just two RI bipartitions (#s 1596 and 1618), followed by ASTRAL_BP analysis of the remaining 2111 RIs, yielded an optimal ASTRAL_BP tree that contradicts nine clades that are each weakly supported by ASTRAL_BP on all 2113 RIs (‘clades a–i’ in Figure 6). If support were completely independent at these nodes, the removal of 13 RI loci would be required to erase all nine clades (i.e., 2 + 2 + 1 + 1 + 1 +2 + 2 +1 + 1 = the sum of locus removal index scores for clades a–i). The dependent support at multiple nodes in the ASTRAL_BP tree was also apparent in ‘anti-constraint’ searches [77] for the best topologies that lacked particular supported clades. For example, the best ASTRAL_BP tree that lacked ‘clade a’ (Telluraves + Aequornithes + crane) also contradicted two additional clades in the ASTRAL_BP tree (‘clades d and h’; Figure 7), and the best tree without ‘clade b’ contradicted three additional clades supported by the optimal ASTRAL_BP tree (‘clades c, d, and h’; Figure 7). Such ‘linked’ branch support among nodes [81,92,93] indicates that the overall stability of a phylogenetic hypothesis is even lower than indicated by the isolated support scores at individual nodes in the species tree.

Despite congruence with trees supported by some independent phylogenomic analyses [1,2,82], by all measures, the intriguing basal split between Strisores and all other species of Neoaves was exceptionally weakly supported by the ASTRAL_BP analysis of RI bipartitions (Figure 4, Figure 5, Figure 6 and Figure 7). The branch length that subtends this clade was estimated at 0.000 CUs (implying a polytomy), the Bayesian local PP was just 0.13, bootstrap support was also extremely low (21%), and the removal of only one RI bipartition was enough to collapse this clade. The effective number of loci (EN) is a critical measure that must be considered when assessing the support and branch lengths in ASTRAL_BP analyses of RGCs [60]. EN was only 32 for the clade of all Neoaves except Strisores, indicating that very few RI bipartitions are informative (i.e., provide discriminating quartets) at this node in ASTRAL_BP analysis. EN was also relatively small, ranging from 31 to 82, for the remaining nine low-support clades at the base of Neoaves that generally conflicted with previous phylogenomic work (Figure 4). The low ENs at these critical nodes are determined by several factors. Most importantly, each RI character supports only a single bipartition of taxa, in contrast to sequence-based gene trees, wherein every resolved bipartition (clade) in the gene tree provides phylogenetic information (unrooted quartets) in a standard ASTRAL analysis. Even though the RI dataset was large (2113 total characters), a relatively small subset of RI bipartitions had a bearing on the deepest divergences in Neoaves (Figure 4). Missing data (mean of 28% per species; Table 1) also reduced the number of informative RI characters in introgression tests that require complete sampling [56,59,68], and more generally, uneven sampling of taxa can influence the relative weights of some loci versus others in quartet-based coalescent analyses [31]. Higher quality genome assemblies for the major extant lineages of birds [94] may help to fill in some of these missing data in the current RI matrix [5] and also permit expanded coding of many more RGC characters in the future.

### 4.4. Hidden (Emergent) Support

Hidden (emergent) support was recorded for three clades in the quartet-based coalescent tree. Specifically, three nodes (clades a, b, and d; Figure 6) that were supported by ASTRAL_BP and SDPquartets (Figure 4) are not corroborated by even a single perfectly congruent RI synapomorphy (Figure 8B). This indicates that these three nodes are completely emergent and are each supported in quartet-based coalescent analysis only by conflicting RI loci characterized by ILS, introgression, and/or homoplasy. Such hidden support in summary coalescent analyses has been noted previously for contrived [25] and simulated [78] datasets, and our RI species tree represents an empirical example of this phenomenon (Figure 8B). All three of these emergent clades (a, b, and d) are unstable to the removal of just two RI bipartitions (#s 1596 and 1618). Like all other RI bipartitions in the dataset that are informative at these three emergent nodes, bipartitions #1596 and #1618 conflicted with and did not cleanly support clades a, b, and d, despite their strong influence in resolving these emergent nodes. In fact, none of the nine clades that collapsed with the removal of bipartitions #1596 and #1618 were cleanly supported by either of these two RI loci (Figure 6).

Because each RI character can be represented as a single bipartition in an otherwise unresolved gene tree (e.g., Figure 6), RIs can be merged with standard sequence-based gene trees in more comprehensive combined ASTRAL coalescent analyses that incorporate information from diverse genomic resources. In our equally weighted ‘total evidence’ coalescent analysis of >6000 DNA-sequence-based gene trees (introns and UCEs) with 2113 RI characters, the RI characters impacted the species tree based on just the DNA-sequence data (Figure 8A). Two unique clades emerged in the total evidence ASTRAL tree. Within Telluraves, a Cavitaves + Coliiformes + Strigiformes clade and a Cavitaves + Coliiformes + Strigiformes + Australaves clade were supported (Figure 8C). The overall topology for Telluraves matched several previous genome-scale concatenation [2] and binned-coalescent [1] trees (Figure 2B). Bayesian local PPs were high at these nodes (Figure 8C), but perfectly congruent RIs that supported these emergent relationships (‘hidden synapomorphies’) were few relative to the RI support for three conflicting clades in the ASTRAL_BP tree based on just RIs (Figure 8B). It is noteworthy that two robustly supported clades in the DNA-sequence-based tree (local PPs = 1.0) were overturned by the addition of the comparatively small RI dataset and that the two emergent clades in the total evidence tree had high support scores (local PPs = 0.95, 1.0). At other nodes in the combined-data species tree that conflicted with the RI tree, local PPs were not impacted greatly by the addition of RI bipartitions to sequence-based gene trees for introns and UCEs (Figure 8).

In the equally weighted combined ASTRAL analysis, 18 perfectly congruent RIs emerged as hidden synapomorphies for basal clades supported by the total evidence species tree (Figure 8C) that conflicted with the ASTRAL_BP tree based on just RIs (Figure 8B). This emergent RI support at controversial deep nodes provides further corroborating evidence for an initial split in Neoaves between Columbea (3 loci) and Passerea (six loci) as well as additional support for Phaethoquornithes (five loci), Otidimorphae (three loci), and Columbimorphae (one locus), clades that are part of an emerging phylogenetic consensus for Neoaves (Figure 1). The hidden support for relationships that are not resolved by separate analysis of the RI data (Figure 8B) hints at additional congruence between data types that may be accentuated if still larger RGC datasets with less missing data are generated from more complete avian genome assemblies in the future [94]. In contrast to Suh [6], Reddy et al. [3] and Houde et al. [8] argued that the base of Neoaves may not be a ‘hard polytomy’ because some of the earliest branching events showed congruence between both coalescent and concatenation analyses of genome-scale datasets. Combined ASTRAL analysis of RIs and sequence-based gene trees (Figure 8C) additionally revealed a hidden RI signal for these same basal clades (e.g., Passerea, Columbea, Otidimorphae).

### 4.5. Weighted ASTRAL Analyses and the Phylogenetic Placement of Mousebird

‘Total evidence’ coalescent analyses of >2000 RIs plus >6000 gene trees recorded striking changes in both the topology and clade support within Telluraves. More severe downweighting of poorly supported gene tree nodes (9B–D) yielded higher congruence with the ASTRAL_BP species tree for RIs (Figure 9A). In particular, the high local PPs for mousebird + Cavitaves (Figure 8A and Figure 9B,C) were decisively overturned in combined wASTRAL analysis when gene tree nodes with <50% bootstrap were collapsed, nodes with 50–100% bootstrap were weighted differentially, and RI bipartitions were given the maximum weight (100). This combined wASTRAL result (Figure 9D) implies that the seemingly robust support (local PP = 1.0) for mousebird + Cavitaves in the ASTRAL analysis of 2516 introns and 3679 UCEs (Figure 8A) is dependent upon the most unreliable nodes in sequence-based gene trees.

### 4.6. Evidence for Introgressive Hybridization in Afroaves?

Braun et al. [4] neatly summarized the difficulties in discerning introgression/hybridization in bird phylogeny, especially deep in the avian tree. They noted that (p. 186), “If we focus deeper in evolutionary history, the impact of hybridization is expected to be more difficult to examine: gene tree estimation error might make it virtually impossible to distinguish the descendants of lineages that underwent limited amounts of ancient hybridization from those with purely treelike history (combined with ILS).” The MSC predicts that the two minority resolutions at a supported node should have equal frequencies (in terms of unrooted quartet or rooted triplet support), within the sampling error, if gene tree conflicts are simply due to ILS at a node [65,95], but Braun et al. [4] correctly recognized that “…errors in estimated gene trees can either produce or obscure inequalities in the numbers of gene trees with each minority resolution, limiting the utility of the inequality test.”…“Developing practical tests that are able to establish whether the null hypothesis of ILS alone can explain discordance among gene trees represents a major challenge in the phylogenomic era.” Because RI characters are expected to have much lower homoplasy than nucleotide substitutions in a gene sequence, especially for comparisons among deeply divergent lineages (e.g., Figure 5), such RGCs might be especially useful for distinguishing gene flow signals from ILS [52,56,59,68].

Introgressive hybridization [5,6] offers another possible explanation for the scattered phylogenetic results within Telluraves. Alternative species trees commonly express robust conflicts with no strong consensus among concatenation and coalescent results (Figure 2). Indeed, this is precisely what we observed in separate versus combined coalescent analyses of RIs and sequence-based gene trees (Figure 8 and Figure 9). Houde et al. ([8]; their Figure 7) recorded highly skewed quartet frequencies at several deep nodes in Afroaves (e.g., Strigiformes + Accipitriformes) for thousands of gene trees based on UCEs and introns, but due to gene tree reconstruction error, these asymmetries do not necessarily provide strong evidence for introgression in Telluraves. Multiple factors such as convergent base compositional shifts [3] or long-branch misplacement [96] can bias gene trees toward favoring one minority resolution over another. For example, Simmons et al. [13] recently suggested that misrooting by long branch attraction between a divergent outgroup and rapidly evolving ingroup lineages likely generated quartet asymmetries at deep nodes in Palaeognathae (ratites and tinamous), and analogous rooting irregularities that bias phylogenetic interpretations have been noted in prior genome-scale studies [28,97].

The detailed examination of RIs in [5] did not reveal any strong introgression signals involving the mousebird lineage, but possible RI asymmetries have been noted for relationships among Strigiformes, Accipitriformes, and Cavitaves based on an earlier version [1] of the RI dataset reanalyzed here [5]. For the full dataset of 48 taxa and 2113 informative RIs, skewed RI support for minority resolutions at the controversial Strigiformes + Cavitaves node within Afroaves was detected using several measures (Figure 10). This asymmetry was expressed in Bayesian local PPs (Figure 10A), perfectly congruent RI synapomorphies (Figure 10A,C), PCS scores (Figure 10B,C), and quartet frequencies (Figure 10C). In all cases, these related indices showed an excess of support for Strigiformes + Accipitriformes relative to Cavitaves + Accipitriformes. Such asymmetries between the two minority resolutions at a node hint at possible introgression, but in the context of ILS, it is a challenge to determine whether introgressive hybridization has generated these skewed patterns for the complete dataset of 48 taxa.

Subanalyses on smaller sets of taxa, those with the least missing data (Table 1), were conducted to statistically test for significant skew, which is indicative of introgression in the Cavitaves + Strigiformes + Accipitriformes clade. For the subset of taxa in Afroaves with least missing data for major lineages (owl, cuckoo-roller, eagle 2, mousebird) and an outgroup (seriema), the quartet asymmetry test did not imply significant asymmetry for any of the five possible species-quartets. The most skewed species-quartet (owl, cuckoo-roller, eagle 2, seriema) approached significance (*p* = 0.0817). The two minority resolutions had 40 supporting RIs ((owl, eagle 2)(cuckoo-roller, seriema)) versus only 25 supporting RIs ((eagle 2, cuckoo-roller)(owl, seriema)), with 44 RIs supporting the majority resolution that is compatible with the overall ASTRAL_BP tree ((owl, cuckoo-roller)(eagle 2, seriema)). For KKSC, the analysis of owl, cuckoo-roller, and eagle 2 revealed significant RI support for a network (Figure 10D), and 4-LIN also favored a network for one of two four-species subsets that we examined (owl, cuckoo-roller, eagle 2, seriema) (10E). Both approaches imply introgressive hybridization involving the owl (Strigiformes), and the 4-LIN likelihood-based approach estimated approximately equal genetic contributions from cuckoo-roller (55%) and eagle 2 (45%) (Figure 10E). KKSC and 4-LIN generally utilize more RI data than quartet asymmetry tests because RI characters in which just one taxon is coded as absent provide relevant phylogenetic information for KKSC [59] and 4-LIN [68], but are uninformative for unrooted quartets [56].

For the full dataset of 48 taxa, parsimony optimizations of RI characters that support the grouping of owl with cuckoo-roller and those that group owl with eagle 2 suggest that evidence for introgressive hybridization is mostly concentrated very deep in afroavian phylogenetic history. Many RIs that link owl (Strigiformes) to cuckoo-roller (Cavitaves) are also shared by other members of Cavitaves (trogon, hornbill, bee-eater, woodpecker), and many RIs that link owl to eagle 2 (Accipitriformes) are also shared by other members of Accipitriformes (eagle 1, NW vulture). The overall pattern implies that if introgressive hybridization involving Strigiformes is correct (Figure 10D,E), gene flow may have occurred between branches on the stem lineage of Cavitaves and on the stem lineage of Accipitriformes. For the time tree of Jarvis et al. ([1]; their Figure 1), crown Cavitaves and crown Accipitriformes both extend back to the Paleocene (>56 MY), providing an upper-bound estimate on when introgression might have occurred in Afroaves. Such gene flow may explain some of the strong conflicts among phylogenomic analyses that have assumed a strictly bifurcating phylogenetic process (Figure 2).

To our knowledge, the possible hybrid origin of an avian order has not been seriously proposed in previous systematic work. Moving forward, the morphological and behavioral traits of owls, which broadly express both raptorial features and cavity-nesting behaviors, should be considered within the context of various ancient-introgressive-hybridization scenarios if more comprehensive analyses of RGCs and independent data corroborate the KKSC and 4-LIN networks based on RIs (Figure 10D,E). For example, it is intriguing that combined wASTRAL analysis of RIs, UCEs, and introns with strong downweighting of low-support gene tree nodes (Figure 9D) robustly supported a species tree that conflicts at just one internode with our ASTRAL_BP analysis of RIs (Figure 9A). The single difference was the placement of Strigiformes with Accipitriformes instead of with Cavitaves (Figure 9A,D).

### 4.7. Estimating Large Species Trees with RIs: Prospects and Challenges

Resolving ancient rapid radiations is a major challenge for the field of phylogenomics because supermatrix (concatenation) methods do not explicitly account for ILS that is expected to be rampant in such speciation scenarios [23]. Summary (shortcut) coalescence methods that are based on the MSC are statistically consistent when evolution is completely neutral, loci are not closely linked, recombination within loci is absent, and the reconstruction of gene tree topologies is accurate (e.g., [98,99]), but we contend that these assumptions likely do not hold for any genome-scale dataset composed of DNA-sequence alignments [25,26,27]. In particular, gene tree reconstruction error is expected to be extremely high when terminal branch lengths are long, internal branch lengths are extremely short, the informative variation in genes is low, evolutionary constraints on DNA sequences shift among lineages over time, and/or nodes are arbitrarily resolved [3,25,38,43]. RIs represent one possible solution to resolving the Neoaves radiation and similarly intransigent phylogenetic problems [51,52]. These RGCs are characterized by low homoplasy, are not strongly impacted by within locus recombination, and generally occur in genomic ‘safe havens’ that may better approximate neutral MSC conditions in comparison to commonly used phylogenetic markers such as UCEs and protein-coding exons with complex selective constraints [59].

Here, we utilized quartet-based coalescent methods that can be applied to large sets of taxa [56] and approaches for distinguishing deep coalescence from introgression using RIs [56,59,68] in analyses of large datasets that span the early radiation of Neoaves [5]. Recent simulations of RIs [60] hint that quartet-based summary coalescent analyses might be effective for resolving such rapid diversifications that are relatively deep in time. There are several caveats, however, that are critical to consider when analyzing RIs using quartet-based summary coalescent methods. Most importantly, even for very large RI datasets such as the one reanalyzed here (2113 characters), relatively few RI bipartitions might be informative at any particular node in the species tree. EN (Figure 4) at each supported node is therefore a critical measure that should be referenced when interpreting clade supports and branch lengths in the inferred RI species tree [60]. A further complication revealed in the present study is that RI-bipartition support in ASTRAL_BP analysis can be dependent across multiple nodes in a species tree, particularly when ILS has been rampant and coalescent branch lengths are extremely short. Such ‘linked support’ across multiple nodes complicates the interpretations of EN and clade support (Figure 6 and Figure 7), as do missing data that are not randomly distributed across taxa (Table 1) or loci (Figure 8C).

A critical contrast between sequence-based gene trees and RI bipartitions in genome-scale analyses relates to data quantity versus data quality. In the present study, data quantity was greater for sequence-based gene trees because each gene tree provided phylogenetically informative quartets at many nodes in the species tree, while each RI character resolved just one bipartition and was therefore relevant at only one or a few nodes. On average, data quality is presumably higher for low-homoplasy RIs relative to sequence-based gene trees, but whether higher quality generally wins out over greater quantity when reconstructing relationships in a rapidly radiating clade is a topic that requires more study. The quartet-based ASTRAL approach permits coherent ‘total evidence’ coalescent analysis of both RIs and sequence-based gene trees. Such analyses enable the detection of common phylogenetic signals and simultaneous inference of the species tree based on all relevant genomic data (Figure 8C). Combined wASTRAL analyses with differential weighting of low-homoplasy RIs (high weight) versus arbitrarily resolved nodes in sequence-based gene trees (zero weight) versus nodes supported at various levels in gene trees (low to medium to high weights) improved congruence within Afroaves in our study (Figure 9), and initial simulations using the wASTRAL method are promising [66,74]. We are optimistic that with the accurate assembly of even larger RI datasets from more complete genome assemblies and the continued development of quartet-based methods that accommodate both ILS and introgression, it may be possible to resolve even the most challenging phylogenetic questions, particularly rapid radiations that are deep in time (Figure 5) wherein hybrid gene flow may have occurred (Figure 10D,E).

## 5. Conclusions

(a) Overall, there was very limited congruence for relationships at the base of Neoaves between the quartet-based coalescent tree for RIs, polymorphism parsimony analysis of the RI dataset, and previously published genome-scale analyses of DNA sequence data (Figure 4).
(b) Quartet-based RI branch lengths in CUs may be an accurate indication of the extremely challenging phylogenetic problem at the base of Neoaves. Extensive conflicts among RIs imply numerous internal branch lengths that are remarkably short within this deep radiation of avian orders (Figure 4 and Figure 5).
(c) Quartet-based analysis of RIs enables calculation of multiple clade support indices within a coalescent framework (Figure 4), all of which suggest that support at the base of Neoaves is a ‘house of cards’, with interdependence of support scores across multiple nodes (Figure 6 and Figure 7).
(d) Evolutionary constraints on RIs are very different from those on DNA sequences, so it is intriguing that the basal split in our quartet-based species tree for Neoaves is congruent with several previous phylogenomic analyses, but RI support is extremely weak for the controversial clade that includes all Neoaves except Strisores (Figure 4).
(e) Hidden support was noted at three completely emergent nodes in our quartet-based coalescent tree based on RIs (Figure 8B), and 18 ‘hidden RI synapomorphies’ for controversial clades in an emerging consensus of neoavian relationships (Figure 1) were revealed in an equally-weighted combined analysis of >6000 sequence-based gene trees with 2113 RI characters (Figure 8C).
(f) Combined (‘total evidence’) ASTRAL analysis represents a general coalescent framework for inferring species trees from diverse molecular data including sequence-based gene trees [1,2], indels [8,100], RIs [48], chromosomal rearrangements [101], NUMT insertions [102], and other RGCs [47].
(g) Combined wASTRAL analysis [74] enables the upweighting of low-homoplasy RGCs and robustly supported gene tree nodes relative to weakly supported gene tree nodes, which can profoundly impact phylogenomic inference and congruence (Figure 9).
(h) The analysis of RIs in a coalescent framework has great promise for detecting introgressive hybridization that is deep in the Tree of Life due to much lower homoplasy relative to standard DNA-sequence data. For the KKSC and 4-LIN tests, RIs support ancient introgressive hybridization for the owl lineage (Strigiformes), with 55% genetic contribution from Cavitaves and 45% from Accipitriformes (Figure 10D,E). Quartet asymmetry tests did not detect a significant deviation from the MSC in Afroaves.
(i) Improved genome assemblies from many more species of birds [94] should increase the number of informative RIs, reduce missing data (Table 1), and advance phylogenomic inference at the base of Neoaves and within Telluraves.
(j) Future simulations of RIs as phylogenetic characters should assess the impacts of missing data, differential insertion rates, inaccurate character coding, and other factors that might hinder the reliability of quartet-based coalescent methods [60].

## Figures and Tables

**Table 1:** Missing data in the RI matrix of Suh et al. [5]. The number of characters coded as missing (?) out of 2118 total is shown for each species. The average number of missing characters per species was 600 (28% of the total). Species names, common names, and species abbreviations in datasets are given.

Missing Data	Species Name	Name in Trees	Species Abbreviation
885	*Acanthisitta chloris*	passerine 1	acan
1201	*Anas platyrhynchos*	outgroup 1	anas
700	*Apaloderma vittatum*	trogon	apal
80	*Aptenodytes forsteri*	penguin 1	apte
242	*Balearica regulorum*	stork	bale
715	*Buceros rhinoceros*	hornbill	buce
1072	*Calypte anna*	hummingbird	caly
431	*Antrostomus carolinensis*	nightjar	capr
229	*Cariama cristata*	seriema	cari
206	*Cathartes aura*	NW vulture	cath
886	*Chaetura pelagica*	swift	chae
205	*Charadrius vociferus*	killdeer	char
409	*Chlamydotis macqueenii*	bustard	chla
886	*Colius striatus*	mousebird	coli
557	*Columba livia*	pigeon	colu
907	*Corvus brachyrhynchos*	passerine 2	corv
731	*Cuculus canorus*	cuckoo	cucu
213	*Egretta garzetta*	egret	egre
585	*Eurypyga helias*	sunbittern	eury
290	*Falco peregrinus*	falcon	falc
184	*Fulmarus glacialis*	fulmar	fulm
1547	*Gallus gallus*	outgroup 2	gall
184	*Gavia stellata*	loon	gavi
1101	*Geospiza fortis*	passerine 3	geos
162	*Haliaeetus albicilla*	eagle 1	hala
135	*Haliaeetus leucocephalus*	eagle 2	hall
270	*Leptosomus discolor*	cuckoo-roller	lept
833	*Manacus vitellinus*	passerine 4	mana
1644	*Meleagris gallopavo*	outgroup 3	mele
780	*Melopsittacus undulatus*	parrot 1	melo
746	*Merops nubicus*	bee-eater	mero
596	*Mesitornis unicolor*	mesite	mesi
679	*Nestor notabilis*	parrot 2	nest
128	*Nipponia nippon*	ibis	nipp
311	*Opisthocomus hoazin*	hoatzin	opis
164	*Pelecanus crispus*	pelican	pele
278	*Phaethon lepturus*	tropicbird	phae
342	*Phalacrocorax carbo*	cormorant	phal
193	*Phoenicopterus ruber*	flamingo	phoe
1286	*Picoides pubescens*	woodpecker	pico
377	*Podiceps cristatus*	grebe	podi
462	*Pterocles gutturalis*	sandgrouse	pter
88	*Pygoscelis adeliae*	penguin 2	pygo
1317	*Struthio camelus*	outgroup 4	stru
1180	*Taeniopygia guttata*	passerine 5	taen
342	*Tauraco erythrolophus*	turaco	taur
1730	*Tinamus guttatus*	outgroup 5	tina
325	*Tyto alba*	owl	tyto

## Supplementary Materials

The following supporting information can be downloaded at: https://figshare.com/account/home#/projects/137739 (accessed on 26 June 2022).
